# A Longitudinal Analysis of the Association Between Subclinical Hearing Loss and Cognition

**DOI:** 10.1093/geroni/igaa057.3301

**Published:** 2020-12-16

**Authors:** Alexandria Irace, Nicole Armstrong, Jennifer Deal, Alexander Chern, Luigi Ferrucci, Frank Lin, Susan Resnick, Justin Golub

**Affiliations:** 1 NewYork-Presbyterian Hospital/Columbia University Irving Medical Center, New York, New York, United States; 2 ^2^ Brown University Warren Alpert Medical School, Providence, Rhode Island, United States; 3 Johns Hopkins Bloomberg School of Public Health, Baltimore, Maryland, United States; 4 National Institute on Aging, Bethesda, Maryland, United States; 5 Johns Hopkins University School of Medicine, Baltimore, Maryland, United States

## Abstract

Several studies have demonstrated that age-related hearing loss (defined as >25 dB pure tone average [PTA]) is longitudinally associated with worse cognition. We aimed to investigate whether subclinical hearing loss (SCHL), or imperfect hearing traditionally categorized as normal (PTA ≤25 dB), may be similarly linked to cognitive decline. Subjects included cognitively normal adults ≥50 years old in the Baltimore Longitudinal Study of Aging with PTA ≤25 dB measured between January 1991 - September 1994 who had repeated cognitive assessments from January 1991 - November 2019 (n=263). The exposure was hearing based on the better ear PTA. The outcomes were standardized test scores in the following domains: learning/memory, mental status, executive function, visuospatial ability, and language. Multivariable linear-mixed effects models with random intercepts and slopes and unstructured variance-covariance structure were used to model the association between hearing and change in cognition over time, adjusting for baseline age, sex, years of education, and race. Mean age was 68.3 years (standard deviation [SD]=8.9) and follow-up ranged from 0-27.7 years (mean=12.5, SD=7.9). A 10-dB worsening in hearing was longitudinally associated with an annual decline of 0.016 SDs (95% confidence interval [CI]: 0.0002, 0.033) in California Verbal Learning Test (CVLT) short-delayed recall, 0.019 SDs (95% CI: 0.002, 0.036) in CVLT long-delayed recall, and 0.017 SDs (95% CI: 0.006, 0.028) in letter fluency after covariate adjustment. Poorer hearing among those with SCHL was associated with steeper declines in memory and verbal fluency scores. This relationship may begin at earlier levels of hearing loss than previously recognized.

